# Exploring Stress and Coping Among Urban African American Adolescents: The Shifting the Lens Study

**Published:** 2006-03-15

**Authors:** Anita Chandra, Ameena Batada

**Affiliations:** RAND; Dr Chandra is also affiliated with The Center for Adolescent Health, Johns Hopkins Bloomberg School of Public Health, Baltimore, Md; The Center for Adolescent Health, Johns Hopkins Bloomberg School of Public Health, Baltimore, Md

## Abstract

**Introduction:**

Stress can have a significant effect on an adolescent's long-term physical and mental well-being. An understanding of the role of unmanaged stress during early adolescence is critical for the prevention of chronic diseases such as depression. The purpose of the Shifting the Lens study was to explore perceptions of stress, sources of social support, and use of coping strategies among urban African American ninth graders.

**Methods:**

A youth-driven, mixed-method approach was used to assess teens' perceptions of stress. During the 2001–2002 school year, teen participants (N = 26) from East Baltimore, Md, completed questionnaires, audio journals, pile-sort activities, and personal social support network maps.

**Results:**

In contrast with existing literature that emphasizes the influence of violence and neighborhood factors on stress among teens, teens prioritized other sources of stress, particularly from school, friends, and family. For support, they relied on different individuals, depending on the source of the stress — friends for romantic relationship stress and family for job, school, and family stress. Sex differences in the coping styles of the participating teens were found. Girls reported more frequent use of support-seeking and active coping strategies than boys.

**Conclusion:**

The use of multiple data collection strategies to explore stress uniquely contributes to our understanding of how one group of teens perceives and copes with stress. Future research should explore stress from the youth perspective in communities that are similar to East Baltimore, Md. In addition, programmatic recommendations include the need for sex-specific stress management activities and education about youth stress for adults. Community participatory translation interventions based on study findings, such as a youth-produced video and a resource guide for youth service providers, were implemented.

## Introduction

Teen stress is a pivotal health issue because of its ability to disrupt an adolescent's capacity to handle the demands of daily life, yet it is often overlooked and is poorly understood. Stress emerges for young people as they enter adolescence, a transition that brings rapid socioemotional changes (1). Teens must confront the challenges of developing healthy relationships with peers, meet the expectations of school and the responsibilities of family, and negotiate life in their neighborhoods. Chronic stress, or stress left unchecked or unmanaged, can have a profound impact on an adolescent's physical and mental well-being, leading to illnesses such as depression (1-4). Understanding the role and impact of stress is an important first step in the prevention and treatment of its associated chronic diseases.

Research on adolescent stress and its relationship with mental and physical health problems has been conducted, but the work is limited in two ways. First, the studies often rely on quantitative methods to identify the sources and impact of youth stress. Although this is a useful way to assess levels of stress, qualitative tools would add a unique perspective on how young people themselves discuss and prioritize issues (1-3,5,6). Second, research on stress among urban minority youth is limited. When researchers have explored stress among them, they have tended to focus on violence and neighborhood variables, and unlike suburban studies, they place less emphasis on other sources of stress (e.g., school) for these teens. More specifically, little is known about how African American teens living in urban settings discuss stress and what they cite as the most significant sources of stress (7-10). Given the rising rates of youth suicide and depression in the African American community and the problems of unmet mental health need, understanding what teens consider to be causes of stress is essential (11,12).

In an attempt to address these limitations, we developed the Shifting the Lens study. The study's goal was to use a youth-driven, mixed-method approach to explore perceptions of stress, sources of social support, and use of coping strategies among urban African American teens.

## Methods

The Shifting the Lens study involved quantitative and qualitative techniques to collect data from teens, their primary caregivers, and youth service providers; in this article, we focus on the data provided by teens. The study was approved by the Johns Hopkins Committee on Human Research, and study design and instruments were reviewed by the Johns Hopkins Center for Adolescent Health Youth Advisory Committee.

### Study population

Twenty-six African American teens who were living in or attending ninth grade in East Baltimore, Md, participated in the study. The East Baltimore community is predominately African American and low income. Many young people live in single-parent households, and most teens either walk or use a city bus for transportation to school.

### Data collection

Researchers used a snowball technique to recruit teens, beginning with an initial group of 10 participants known personally by one of the researchers. Although the goal of the study was to recruit 50 teens (10 in each of five groups), not all of the teens who participated referred other youths to the study; a total of 26 teens in five groups participated. The teens participated for a 1-month period from February 2001 to June 2002 of their ninth-grade year. The data collection techniques included a self-administered questionnaire, a month-long audio journal, a pile-sorting activity for sources of stress, and a personal network map. The researchers used additional data collection and pilot techniques with group 1, the initial group of 10 teens. Group 1 attended a full-day workshop to review the questionnaire and generate information for study instruments.

At the end of the first meeting for each group in which teens completed questionnaires and submitted signed parental consent forms, participants received tape recorders and calendars for the month-long audio journal. At the end of 4 weeks, the group returned to the study site with their tape recorders, completed the remainder of the data collection items, received compensation, and brought a friend for the next group.

### Instruments

#### Questionnaire

The 16-page self-administered questionnaire included questions about demographics, stress symptoms, family conflicts, coping strategies (13), and racial discrimination. Teens from group 1 indicated that the questionnaire was too long, so we shortened it by eliminating the Adolescent Perceived Events Scale (6).

#### Audio journal

The month-long audio journal included a calendar with daily questions to be answered on tape by the teen. Most questions were fillers to maintain daily journal consistency (e.g., "What is your favorite flavor of ice cream?" "To what kind of music do you like to listen?"). Every third day the question related to the study. The topical study questions included items about how the teens defined and perceived stress as well as probes about their experiences of stress and coping at school, with the family, in peer relationships, and in the neighborhood. All questions about teens' experience with stress had a similar structure and included a probe to describe the stress and a probe to discuss their related coping strategy.

The audio journal was not familiar to the teens; however, the approach gave them an opportunity to share their thoughts in their own words without the potential bias introduced by an interviewer. Fidelity to this method was high; most teen participants (25 of 26) completed all the entries in the audio journal. Responses to questions varied in length from about 3 minutes to longer than 10 minutes. On average, teens spent between 5 and 10 minutes on stress-related questions but only 2 or 3 minutes on the filler items.

#### Pile-sort activity

Sixteen sources of stress were identified by group 1 during their group discussion, and the sources were used in the pile-sort activity. Twenty-five teens completed one free pile-sort and two structured pile-sort activities using the 16 source-of-stress cards. First, teens free–pile-sorted the stress cards in any way that made sense to them. Second, the teens sorted the stress cards according to how frequently the source of stress occurred in their lives (never, rarely, sometimes, often). Third, teens sorted stress cards by their level of worry about the sources of stress (no worry, a little worried, somewhat worried, very worried).

#### Personal network map

Using instructions provided by the researchers, teens created personal network maps, or illustrations of the individuals and places they use for support (n = 25). One teen did not complete the second part of the study, which included the audio journal and personal network map. The approach was based on social network analysis methodology to assess types and importance of members in an individual's social support network (14).

### Data analysis

Researchers used SPSS version 11.5 (SPSS Inc, Chicago, Ill) to analyze the quantitative data from the questionnaire. Chi-square and analysis of variance were used to examine bivariate relations by sex. Researchers also used SPSS to calculate frequency data from the pile-sort activities. Research assistants transcribed the text of the audio journals, and researchers individually coded the text in Atlas.ti version 4.2 (Scolari, Berlin, Germany). Interrater reliability was assessed by comparing the transcript coding between the researchers. Inductive coding techniques were used to identify categories and concepts that emerged from the transcripts (15,16). The interview findings were categorized into themes. Exemplary quotes highlighted the salient themes, particularly ideas that were frequently shared by respondents (16).

The researchers interacted personally with each participant in the study, and the findings reflect the data collected using the various study tools, as well as the researchers' experiences and observations during the day spent with group 1 participants and throughout the process of meeting the teen participants. This approach to understanding the data was useful and appropriate given the exploratory nature of the study.

## Results

### Respondent characteristics

The average age of the participants was 14.5 years (SD = 0.7). The majority of the participants were female (19, or 73%). The teens represented nine high schools in East Baltimore, Md; however, 16 (62%) of them attended three high schools. Household size ranged from two to seven or more people. The mean household size was 4.5 people (SD = 1.4), and the average number of children in the households was 2.4 (SD = 1.3). Almost all of the participants (25, or 96%) had at least one sibling. Most of the teens (24, or 92%) lived with at least one of their biological parents, and most participants (22, or 85%) cited a parent as their primary caregiver. The remaining teens (4, or 15%) cited a grandparent as their primary caregiver. In the past year, seven (27%) of the youths had received mental health services such as counseling.

### Definitions of stress

In their audio journals, teens were asked to describe what stress meant to them. Participants offered definitions of stress with great ease and acknowledged that stress can be unhealthy for teens. One teen remarked:

I do think stress is [a] problem for teenagers because when you a teenager, that's the time every move, you know, every thing you do . . . It's like . . . they [parents] don't trust you, or they being too hard on you, or they just want to be in your business . . . but sometimes teenagers need a lot of space because they have a lot of peer pressure and all that.

Teens also discussed that although some of their peers may not use terms such as *stress* in their daily lives, they report having difficulties. One participant explained:

I think teenagers have stress but they really don't know. We have an idea what "stress" mean, but we don't really know what we have in our life is "stress." . . . But teenagers go through a lot with their friends and classmates.

Teens described stress in terms of physical and emotional outcomes. Boys tended to describe physical ramifications of stress, whereas girls tended to focus on physical and emotional consequences. One boy described it as "a great deal of pain that's inside your body that you can't get out and it's agitatin' you and it's stuck in your mind and makes you feel bad." Girls equated stress with feeling anger, frustration, sadness, and physical discomfort. For instance, one participant revealed, "Stress means to me worrying, keeping secrets, gray hair, problems, anger, being tense. Just thinking about somethin' can make you stressed out."

In addition to these definitions, many teens also explained that parents and other adults did not acknowledge their stress. They stated that parents and teachers did not understand the challenges they faced at school and in their relationships.

### Sources and impact of stress

**Figure 1:**
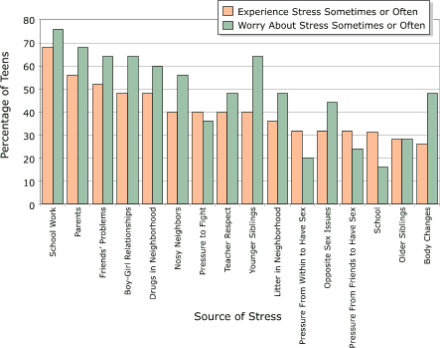
Percentage of teens who reported experiencing or worrying about different sources of stress, Shifting the Lens study.

Quantitative and qualitative data were collected on family stress, peer stress, romantic relationship stress, school stress, and neighborhood stress. Figure 1 shows the percentage of teens who reported experiencing or worrying about different sources of stress.

**Table:** 

#### Family stress

In their responses to the questionnaires, teens noted that family conflicts usually involved doing their homework, cleaning their room, and doing chores. In pile-sort activities, boys (67%) and girls (68%) both noted that parents were a frequent source of stress (*P* = .75); however, more boys (67%) than girls (53%) felt somewhat or very worried about parental stress (*P* = .55).

In the audio journals, teens described enjoyable and stressful experiences in the family context. One source of frequently cited family stress involved worrying about the well-being of family members. One teen commented:

My grandmother, she has a cold now, and I'm worried about her because she has asthma and every time she gets asthma she has real bad colds and stuff. . . . And sometimes a cold can turn into pneumonia. . . . And that's one stressful thing that I always worry about when she gets sick.

Stress involving siblings was a recurrent theme in the audio journals, group 1 sex-specific focus groups, and pile-sort activities. Teens noted that younger siblings were frequent sources of stress, yet boys indicated slightly more worry (50%) about younger sibling stress than girls (37%) (*P* = .57). Some of the sibling stress arose from conflict over family responsibilities or from observations of stressful situations involving their caregivers and siblings. Several boys described feeling stress because of being the only male in the household — having to care for mothers and sisters as well as "defend" the home. One respondent revealed, "Since I'm the only male in my house, it seems like [they] put a lot of responsibility on me to be the responsible male and to protect them [mother and sisters], so that brings a lot of stress on me."

#### Peer stress

In their audio journals, participants described the joys of friendship and social interactions; however, they identified feeling stress from observing friends who were having difficult relationships, dealing with friends' problems, or directly being hurt by a friend. One teen reported:

My friends can give me stress because sometimes I care about most my friends cause, cause well, I just care about them, and they give me a lot of stress because whenever we get in arguments or we get in a fight with somebody, I think that person goin' get hurt or somethin' in the future, and sometimes it just get on my nerves.

The stress over friends' problems was common for boys and girls; however, more girls (58%) cited worry about this source of stress than boys (33%) (*P* = .29).

Many teens described stress from peer pressure and changing peer relationships, particularly during the transition to high school. They explained that this stress was a result of school change and conflicting expectations of friendships. One participant commented:

Most teens go through stress because of peer pressure. They either influenced by something, or their mother or father on drugs, or they got school problems, or people jealous of them. And, teens just have a lot of problems these days because they want to be grown fast.

#### Romantic relationship stress

In the pile-sort activity, more than half of boys and girls cited boy–girl relationships as a frequent (i.e., sometimes or often) cause of stress. On the other hand, more girls (53%) than boys (33%) stated that this type of stress created considerable worry (*P* = .41).

According to their responses to audio journal questions, girls and boys experienced different types of stress from romantic relationships. Among girls, the prevalent stressor involved the honesty of boyfriends: "Romantic relationships can sometimes lead to stress because you fall in love with the person and they stress you out because they cheating on you or because they keep lying to you." Boys commented that their girlfriends constantly questioned them about trust and pressured them to provide material items: "The stress that it brings to me is that I just want to give her the world, and sometimes the stuff that she want I can't get . . . and it makes her look sad but it stress me out to make her think that I ain't got it to give."

#### School stress

School stress was clearly articulated by the majority of teens. In the pile-sort activity, school work was identified as the most frequent and important source of stress. Teens felt stress from the increased amount of homework in the ninth grade and from worrying about exams and grades. More boys (83%) than girls (61%) expressed worry about the amount of school work (*P* = .36). One participant reported, "School cause stress, oh my goodness, so many tests to take, so many classes to go to it give you a headache, it just wouldn't feel right, like all this pressure put on you for just this one little thing that you need in life to get through life and it wasn't right, all these tests."

Many teens also described the stress of adjusting to high school, which included interacting with new people and negotiating a new environment. One teen reported:

High school is way different than middle school. Middle school was . . . it wasn't nothing. Middle wasn't really nothing. High school was a big thing for me, and I always thought that people ain't going like me and I might going have to do this . . . I might going have to do that to fit in.

Several teens discussed stress from teacher relationships, particularly from a perceived lack of respect from teachers as well as general conflicts. Student–teacher relationships were a critical source of stress that youths cited as inhibiting their academic performance and school functioning. One respondent described:

And like sometimes in school, I receive some work that I did better than another person, but the other person got a better grade than me. And I tell the teacher, but she wouldn't really pay it any mind until I had to yell at her, and then she will call my house and I'll get in trouble. I'm dealing with this by trying to stay more humble and notice when I'm yelling.

#### Neighborhood stress

There were some sex differences in the frequency and types of neighborhood stressors. No boys indicated that nosy neighbors were a concern, whereas the majority of girls (74%) experienced this stress (*P* = .004). Whereas boys and girls both cited drugs as a somewhat frequent source of stress in their neighborhoods, far fewer boys (17%) than girls (58%) stated that they were somewhat or very worried about the issue (*P* = .08).

According to audio journals, neighborhood stress came from neighbors who had an unsolicited interest in the teens' lives as well as from drug dealing and litter on the streets. One boy remarked, "Thinking about my neighborhood makes me sick. I really don't like living in the neighborhood that I live in, because everywhere you go is a drug dealer or drug abuser on the corner." Many girls reported concerns about men in their neighborhoods. One girl revealed, "But there's some things that I worry about, like the boys on the next street because they like, older than me. . . . I'm scared to go over in the area because I know that boys that age like to mess you a lot, and I'm not that type of girl. . . . I don't like when boys do that to girls."

Although the teens did feel overall stress from living in their neighborhoods, the sources of stress did not rank as major concerns in the pile-sort activity relative to other sources of stress such as school or parents. Many participants highlighted the positive aspects of their neighborhoods. They discussed feeling happy about and having a sense of calm where they live. As one teen noted, "I like that it's peaceful and quiet, and we have good neighbors, and we don't have a lot of noise going on."

### Sources of social support

**Figure 2:**
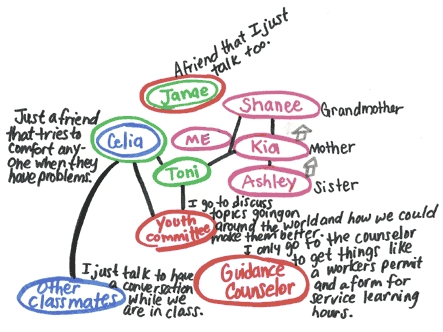
Example of personal network map drawn by a participant in the Shifting the Lens study. (Names have been changed to protect participant identity.)

The personal network maps and audio journals were used to collect data about teens' sources of social support. The personal network maps, which were drawn by the participants, illustrated each teen's perceived network of social support. The maps included people and places such as family members, friends, schools, and communities (Figure 2). The Table contains information about the range, median, and mean number of people and places represented in the personal network maps. Most teens included family members and friends in their personal network map. A review of the personal network maps also revealed that many of the teens drew the circles with family members and friends closer to their own personal circle than the circles with people in other groups (e.g., teachers).

Several teens included people from their school in their personal network maps, individuals such as principals, counselors, teachers, guidance counselors, coaches, and classmates. In the community, teens identified the swimming pool and the Youth Advisory Committee (of The Center for Adolescent Health at Johns Hopkins) as places they used for support. Some participants wrote comments on their personal network maps that explained the reasons they included certain sources of support (e.g., "We like to call each other family," "Encourages me a lot," "Understands me," "Been through a lot").

According to the audio journals, teens relied for support on different individuals depending on the source of their stress. Friends provided support for stress related to peers and romantic relationships. One teen noted the value of her friends: "Talk to my friends, they told me I could do better. They made me feel like I was important. That's how I deal with my stress [with boyfriend] — I talk to my friends and they help me." On the other hand, most teens relied on family rather than friends to discuss challenges from family, school, and jobs. One participant commented, "My stress only comes from school. . . . But I deal with it most of the time with my family — I talk it out."

### Coping with stress

In addition to seeking social support from friends and family members, teen participants used diverse coping strategies to handle their stress. According to their survey responses, the youths reported both avoidant and active coping styles for dealing with the stress in their lives (1). The majority of teens avoided conflicts by trying to stay away from the problem, distracting themselves, or not thinking about the issue. One teen shared in her audio journal, "Sometimes, I'd just close up, not showing feelings to the community or nothin'. . . . I just would blank out from the world and go into my own little place." Many participants also acknowledged trying to talk to a friend or an adult about a problem to figure out how to handle stress; however, most teens did not cope with stress by talking to a nonfamily adult.

**Figure 3:**
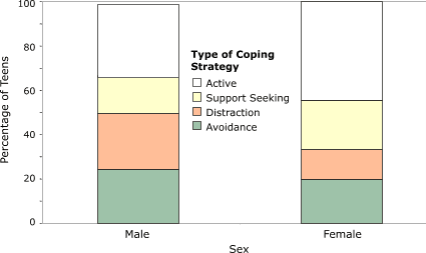
Frequency of coping strategy use, by sex, among participants in the Shifting the Lens study.

There were key differences between boys' and girls' coping styles (Figure 3). A greater percentage of boys reported frequent use (often or most of time) of avoidance (24%) coping skills than girls (20%), and a greater percentage of boys reported frequent use of distraction (25%) coping strategies than girls (14%).

Analysis of variance tests comparing mean number of strategies revealed that boys used fewer support-seeking strategies (2.43) than girls (4.05, *P* = .09) and active strategies (4.71) than girls (8.10, *P* = .04). None of the boys indicated that they generally thought about a problem to handle a situation, whereas almost half of girls did. About one third of girls said that they used prevention strategies such as trying to avoid problems before they developed; however, none of the boys reported that they tried to prevent problems in this way. Some teenage girls wrote down their feelings, whereas none of the boys indicated using this as a coping strategy. Boys were more likely than girls to use sports as a coping mechanism.

**Table:** 

## Discussion

The findings from the Shifting the Lens study are preliminary yet offer critical information about the ways that teens in one urban setting discuss the stress in their lives. The use of multiple data collection strategies to explore stress uniquely contributes to our understanding of how this group of teens perceives and copes with their stress. The insight gained from the study can inform new directions for adolescent stress research and program development.

As mentioned previously, limited research is available on the way African American teens living in urban communities perceive stress. Whereas the results from this study are based on a small sample, the results suggest several future steps for research, particularly with this population. Previous studies of this population have focused on the impact of violence and other neighborhood factors. Given the emphasis that participants placed on school and relationship stress, a more in-depth exploration of how teens cope with these issues is merited. For instance, teens mentioned a lack of respect from teachers. Future research should focus on interactions between teachers and teens so that we can better understand the consequential stress and determine how it can be minimized. Efforts to help teachers understand the sources of stress for teens also may be beneficial for improving the way teachers communicate with their students. This study was conducted with ninth-grade adolescents because early adolescence is a time of accelerated change and stress and thus an important time for intervention. New research should use longitudinal methods to examine how sources of stress may change over the course of adolescence for this population.

Our findings can be used to enhance teen stress programming. First, sex differences in the source and type of anxiety created from certain stressors shows that certain activities in youth stress management programs should be tailored by sex. For example, a focus on the stress from difficulties with authority should be an emphasis for boys. Second, the study findings indicate that teens feel their parents and other adults do not acknowledge their stress. Programs to address teen stress should include education for adults who interact closely with adolescents. The education should consist of information about how teens discuss their major sources of stress. Given the finding that family members are key sources of social support, youth development programs should provide teens with tools they can use to initiate conversations about stress with primary caregivers.

As an extension of this study, three intervention components were developed to apply our research findings in Baltimore City (17). First, in recognition of the need for health education tools that reflect youth perspectives, study participants and other youths translated study findings into a health promotion video about teen stress. The video, *Focus on Teens* (available from www.jhsph.edu/adolescenthealth/Products/Media/focus_medium.mov)*,* was a way for teens to frame and lead a discussion on this topic. The youths created the entire video — from script writing to film editing — and led discussions with groups of local community members, parents, and health advocates. The complete results from the evaluation of the youth-produced video intervention will be presented elsewhere; however, evaluations distributed to community members who attended the screenings and discussions with teens indicate that adults are interested in youth perceptions and input, which have been missing in current research on adolescent stress. Second, the process of creating the video was documented in a guide and video for other researchers interested in engaging youth in participatory approaches to translation of their study findings (available from www.jhsph.edu/adolescenthealth/Products/Media/camera_medium.mov). Third, The Center for Adolescent Health at Johns Hopkins collaborated with community members to produce a practical guide for adults to assist teens with stress. *Confronting Teen Stress: Meeting the Challenge in Baltimore City* (available from www.jhsph.edu/adolescenthealth/Products/Publications/other%20Publications/confronting%20stress.pdf) provides information about teen stress and the Shifting the Lens study and includes stress management activities to use with teens (e.g., one-to-one, small group, large group). More information about this study is available from The Center for Adolescent Health (Johns Hopkins Bloomberg School of Public Health) Web site: www.jhsph.edu/adolescenthealth.

This study has some limitations. As mentioned previously, this study intentionally included a small number of participants. Having fewer participants allowed us to more intensively examine the topic and incorporate less frequently used data collection methods such as the audio journal. Our intention was to include 50 teens, but we were only able to recruit 26. One reason we had difficulty recruiting using the snowball sampling method was that the teens had to complete several consent forms before entering the study — a challenge because we could not meet the teens in groups 2 through 5 before the forms were completed. Our recruitment challenges may have introduced a degree of selection bias. Teens who knew the researchers or who were willing to read and ask their parents to sign the consent forms may have been qualitatively different from the general population of African American teenagers living or going to school in East Baltimore. It is possible that they were more organized (e.g., completing the study paperwork) and perhaps more able to cope with stress than teens who did not participate.

We also recruited fewer boys than girls. Our sample size and unequal number of boys and girls limit the conclusions that can be drawn and generalizability of the study, specifically in terms of statistical significance from the quantitative data. To address the sex disproportion, a concerted effort was made to reflect the boys' perspectives in the analysis and summary of study findings, particularly those obtained from the audio journal. A methodological issue was a limitation as well. To aid teens with the audio journal, we constructed stress categories. The categories were created after obtaining feedback from an initial focus group with teens; however, our categorization may have prevented participants from discussing stress that was not included in these groupings.

The Shifting the Lens study suggests that proximal sources of stress, such as relationships and school, are as important to understand for urban African American teens as more distal sources, such as violence and drugs in the neighborhood. The teens in East Baltimore live in environments that are similar to other minority urban communities in the United States (e.g., Philadelphia, Pa; Washington, DC; Detroit, Mich), in which neighborhoods are predominantly racially homogeneous and generally characterized by limited educational and economic opportunities. The accelerated adolescent experience, in which young people have a compressed childhood because they assume adult responsibilities at a young age, often is typical in these communities (18). Thus, many of these study findings can be applied to the lives of young people in similar urban settings. Future research should explore stress from the youth perspective in communities that are similar to East Baltimore. In this pilot study, teen perspectives were an important component in the development of innovative interventions to address the stress of teens in Baltimore City and other urban communities.

## Figures and Tables

**Table:** Number of People and Places Cited in Personal Network Maps (n = 25), by Group, Shifting the Lens Study

**Group**	**Range**	**Median**	**Mean**
Family	1-9	3	3.6
Friends	1-9	3	3.2
School	0-5	2	1.8
Community	0-4	1	0.9
Other	0-4	0	0.8
